# Thermal damage of internal thoracic artery and ultra high radiofrequency

**DOI:** 10.1186/1749-8090-8-S1-P113

**Published:** 2013-09-11

**Authors:** C Bulat, D Orsulić, Z Marović, M Milić, D Nenadić, J Vojković, V Ćorić, M Carev, N Karanović, D Letica

**Affiliations:** 1 Department of Cardiac Surgery, University Hospital Centre Split, Split, Croatia; 2 Department of Anesthesiology, University Hospital Centre Split, Split, Croatia

## Background

The gold standard in left anterior descending branch coronary artery surgery is performing an internal thoracic artery (ITA) bypass. Skeletonization techniques of harvesting have been suggested for maximizing the utility of ITA. It still remains questionable what is the best method for ITA harvesting in a skeletonized fashion according to structural integrity of artery, as a risk factor of early and late graft failure. The purpose of this study was to determine the impact of the ultra-high radiofrequency energy (2.0 – 4.0 MHz) used for ITA harvesting on arterial structural integrity, in particular on the endothelial layer.

## Methods

Seventy-four ITA specimens were divided into two groups depending on device used for harvesting (radiofrequency-knife (RF) or electrocauter (EC)). Thermal damage on arterial structural integrity was measured using light microscope, morphometric imaging analysis and immunohistochemical methods.

## Results

Thermal damage of endothelium was 2.8 times higher in EC than in RF group (p=0.041) and 5 times higher in patients older than 66 years of age (p=0.002). Extent of endothelial damage (graded from 0 to 3) was significantly higher in EC group (p=0.03).

## Conclusion

The endothelial damage was more often in EC than in RF group as in the patients older than 66 years of age. Demonstrated results suggest that the radiosurgery in comparison to conventional electrocautery could reduce thermal damage to the endothelial layer of ITA, compared to the conventional electrocautery and potentially optimize the quality of ITA bypass grafts.

